# Surface Electromyography in Dentistry—Past, Present and Future

**DOI:** 10.3390/jcm13051328

**Published:** 2024-02-26

**Authors:** Grzegorz Zieliński, Piotr Gawda

**Affiliations:** Department of Sports Medicine, Medical University of Lublin, 20-093 Lublin, Poland

**Keywords:** electromyography, electrodiagnosis, temporomandibular joint, history of dentistry, stomatognathic system, masticatory muscles, temporal muscle, electric impedance

## Abstract

Surface electromyography (sEMG) is a technique for measuring and analyzing the electrical signals of muscle activity using electrodes placed on the skin’s surface. The aim of this paper was to outline the history of the development and use of surface electromyography in dentistry, to show where research and technical solutions relating to surface electromyography currently lie, and to make recommendations for further research. sEMG is a diagnostic technique that has found significant application in dentistry. The historical section discusses the evolution of sEMG methods and equipment, highlighting how technological advances have influenced the accuracy and applicability of this method in dentistry. The need for standardization of musculoskeletal testing methodology is highlighted and the needed increased technical capabilities of sEMG equipment and the ability to specify parameters (e.g., sampling rates, bandwidth). A higher sampling rate (the recommended may be 2000 Hz or higher in masticatory muscles) allows more accurate recording of changes in the signal, which is essential for accurate analysis of muscle function. Bandwidth is one of the key parameters in sEMG research. Bandwidth determines the range of frequencies effectively recorded by the sEMG system (the recommended frequency limits are usually between 20 Hz and 500 Hz in masticatory muscles). In addition, the increased technical capabilities of sEMG equipment and the ability to specify electromyographic parameters demonstrate the need for a detailed description of selected parameters in the methodological section. This is necessary to maintain the reproducibility of sEMG testing. More high-quality clinical trials are needed in the future.

## 1. Introduction

Surface electromyography (sEMG) is a technique for measuring (development and recording) and analyzing the electrical signals of muscle activity using electrodes placed on the skin’s surface [1,2]. sEMG works by detecting and analyzing electrical signals that result from physiological changes in the cell membranes of muscle fibers [1]. A key aspect of sEMG is the understanding that human tissue, particularly muscle, has the ability to generate and conduct electrical impulses that are fundamental to the process of muscle contraction [3]. When a muscle is at rest, it is in a state of electrical equilibrium known as the resting potential. However, during contraction, depolarization of the muscle membrane occurs, which means that there is a flow of ions between the inside and outside of the muscle membrane, generating an electrical signal that is recorded [1,4,5].

In the field of dentistry, sEMG has become a valuable tool for assessing muscle bioelectrical activity during physiological and parafunctional activities [6], it is also used in the diagnosis of temporomandibular joint and masticatory muscle function and treatment of temporomandibular disorders (TMDs) [7,8,9,10]. Studies on the influence of psychological [11,12,13,14] and physical state on changes in the bioelectrical activity of the masticatory muscles appear on sEMG [15,16,17,18]. The usefulness of sEMG has also been demonstrated in orthodontics, primarily in the monitoring of therapy. Recordings of the bioelectrical signal from the masticatory muscles can also help to assess the effectiveness of interdisciplinary orthodontic–surgical procedures aimed, among other things, at improving the function of the stomatognathic system [19]. Some authors refer to sEMG as the ‘’gold standard” for the examination of the masticatory muscles at rest and in function [20]. Studies confirming the effectiveness of sEMG in dentistry have been appearing systematically for years [6,20,21,22,23]. 

sEMG support has been used in innovative ways in dentistry, for example, in a study by Mapelli et al., in which they evaluated the reorganization of muscle activity in patients with chronic TMDs [24]. The authors showed that chronic TMDs showed functional alterations in their masticatory muscles. Mapelli et al. noted that the changes involved a re-organized activity, mainly resulting in worse co-ordination during maximal voluntary contraction and increased participation of balancing side muscles during chewing function [24]. Another study which may be an example of the use of sEMG in orthodontics is a study conducted by Saccucci et al. [25]. The aim of this study was to assess by sEMG the changes in upper and lower orbicular oris muscles produced by a preformed functional device in subjects with malocclusion. The findings of Saccucci et al. suggest that the use of a preformed functional appliance in interceptive orthodontics induces a significant increase in sEMG activity of the orbicular oris muscles at rest and function muscles [25].

The history of surface electromyography in dentistry relates to the origins of anatomy, the search for what moves tissue, the discovery of electricity, detection and analysis of tissue bioimpedance and the history of dental research. One of the first experiments analyzing the activity (strength) of the masticatory muscles took place in 1681 when Giovanni Alfonso Borelli placed a weight under the patient’s mandible [26,27,28]. Most milestones in the field of sEMG in dentistry took place in the 20th century. For example, sEMG for the analysis of the masticatory muscles was used in 1949 by Robert E. Moyers [29]. He was the first to use electromyography to study the masticatory muscles [30]. In 1956, Greenfield and Wyke published a paper in which they determined the placement of surface or needle electrodes when studying the masticatory muscles. This is one of the steps in the reproducibility of electromyographic studies [31]. In 1963, Peter Vig published the first review paper on electromyography in dental science. In this paper, he described the techniques and their limitations in electromyographic studies of mandibular movements [30]. In 2000, another milestone was reached in the form of a surface EMG program for non-invasive muscle assessment (SENIAM) program to standardize sEMG testing [32].

There have been three historical reviews of electromyography (EMG) and sEMG in the 21st century [33,34,35]. Ladegaard’s paper focused on the history of electromyographs since 1950 [35]. Cram’s work focused on sEMG in the 20th century [33]. The more recent review by Kazamel and Warren focused on the 18th century to the present [34]. However, neither review addressed the history of sEMG in dentistry. In addition, none of the reviews covered the history since the 17th century. On the basis of this information, it was decided to write this paper to highlight the history of sEMG in dentistry.

In a historical context, it is also important to highlight the current state of affairs regarding sEMG in dentistry. In the PubMed database, 97,534 records appear under the keyword “surface electromyography” [36]. When the keyword “dentistry” is added, 3136 results appear in the same database [37]. This shows a systematic increase in interest in sEMG in dentistry.

The aim of this paper was to outline the history of the development and use of surface electromyography in dentistry, to show where research and technical solutions relating to surface electromyography currently lie, and to make recommendations for further research.

## 2. Surface Electromyography in Dentistry—History

The history of surface electromyography in dentistry relates to the origins of anatomy, the search for what moves tissue, the discovery of electricity, detection and analysis of tissue bioimpedance and the history of dental research.

### 2.1. The 17th Century

**Figure 1:**
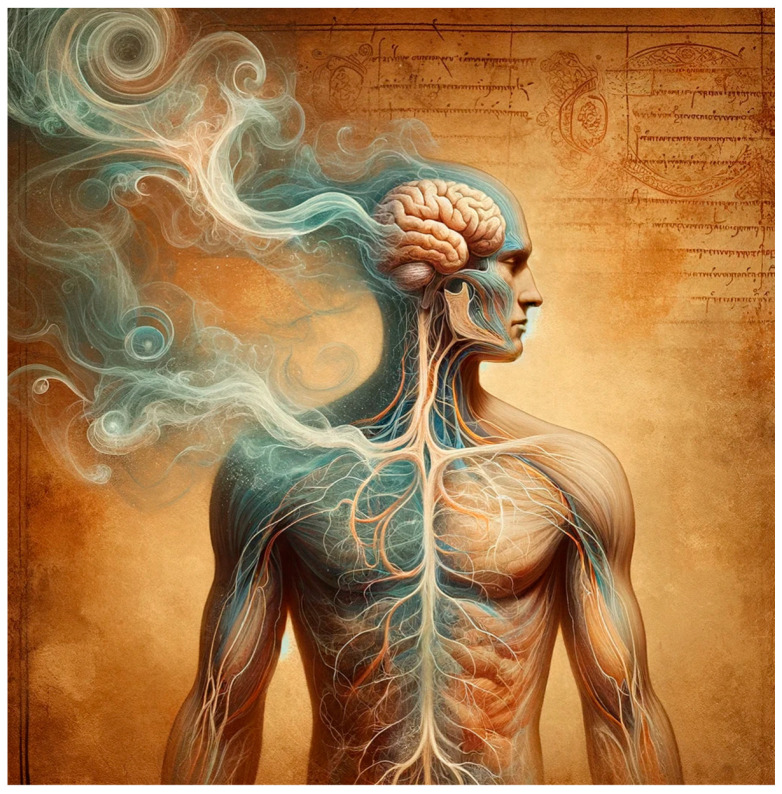
An artistic interpretation of the historical concept of ‘animal spirits’ in human physiology. Derived on 2 January 2024 by DALL-E 2 program (OpenAI, Inc., San Francisco, CA, United States).

The concept of “animal spirits” has a rich history in the realm of physiology and psychology. It was a concept that worked for 1500 years (Figure 1) [38]. Originating in ancient times, it was initially proposed by Greek philosophers like Galen, who believed that these spirits were responsible for sensation and movement. During the Middle Ages, this idea was further developed by Islamic and European scholars, who postulated that animal spirits were fine, vaporous substances flowing through the body’s nerves and brain [39]. People have become increasingly familiar with human anatomy. In 1543, Andreas Vesalius created seven books under the title De Humani Corporis Fabrica Libri Septem on anatomy [40]. However, he still did not know what was responsible for the movement.

In the Renaissance, “animal spirits” evolved with advances in anatomical knowledge, and scholars like René Descartes conceived these “spirits” as liquids or gases, and used the idea to explain reflex action [38,39]. This notion was eventually replaced by more scientific understandings of neurophysiology and psychology, but it played a pivotal role in the early understanding of human bodily functions and mental processes. Jan Swammerdam played a key role in replacing this concept [38]. 

Jan Swammerdam was a pioneer in the study of muscle anatomy and the nervous system, although his work focused primarily on insects and other small organisms. His contribution to the understanding of muscle function was significant, particularly in relation to his experiments on muscle contraction [38,41].

- 1658. Jan Swammerdam showed the Duke of Tuscany how to contract a muscle simply by irritating a nerve [42].
- 1662. Jan Swammerdam, while dissecting dogs, showed that movement could occur without any connection between the muscle and brain, thus putting an end to part of Descartes’ theory [38].
- 1667. Jan Swammerdam proved in his thesis that the diaphragm of a dog could also be moved by stimulating a severed nerve [43]. In the same year, he also proved that muscles do not change their volume during contraction [38].
- 1681. The earliest evidence of research into the strength of the masticatory muscles comes from the work of Giovanni Alfonso Borelli. He recorded a “biting force” of up to 200 kg (about 430 pounds) [26,27]. The test method used by Borelli consisted of placing a weight under the patient’s mandible [28].

### 2.2. The 18th Century

The 18th century was crucial for the development of electricity, marking a transformative period in its understanding and application. This era witnessed the birth of modern electrical science. These advancements paved the way for the electrical innovations of the 19th century, which revolutionized society.

**Figure 2:**
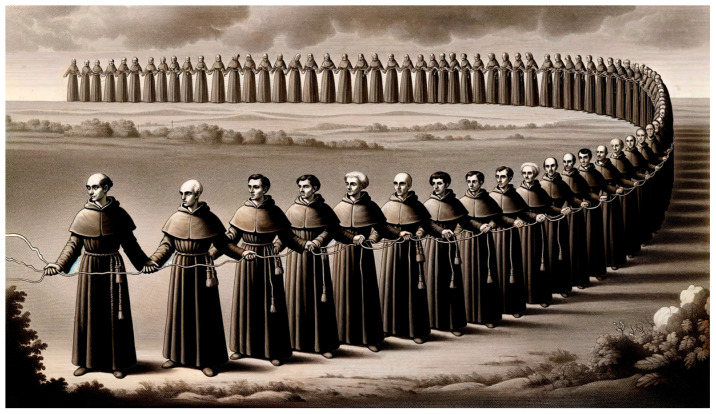
An illustration of Jean-Antoine Nollet’s experiment. Derived on 13 January 2024 by DALL-E 2 program (OpenAI, Inc., San Francisco, CA, United States) (slightly corrected by the author G.Z.).

- 1745–1746. The origins of the development of modern electrophysiology can be traced back to the creation of an early condenser. The Leyden jar was invented independently by Dean von Kleist and Petrus van Musschenbroek. This device was used to collect and store electric charge and was one of the first discoveries in the field of electricity [34,44].
- 1746. Jean-Antoine Nollet conducted an experiment in which the test group consisted of about 200 monks who held hands to form a long chain over a kilometer long (Figure 2). At one end of the chain, Nollet used a Leyden jar to create an electric charge. When the charge was released, it passed through the entire chain of monks. Each of the monks felt a shock as the charge passed through their bodies. The monks’ reactions to the electric shock were instantaneous and simultaneous, demonstrating that electricity can be transmitted through the human body over great distances at tremendous speed. This was the first human study of electricity on such a scale [45].
- 1748. Jean-Antoine Nollet invented the first device to detect and measure the presence of an electric charge. As an early tool for the study of electricity, Nollet’s electroscope played an important role in experiments related to electrostatics [46].
- 1752. Benjamin Franklin conducted the famous kite experiment. This experiment was an important step in the understanding of electricity [47].
- 1772. John Walsh’s research on electric fish, such as the electric eel (torpedo), proved that the shocks given off by these fish were a form of electricity. Using a Leyden jar, a device for storing electric charge, Walsh collected and measured electric discharges, providing direct evidence of the electrical nature of the shocks; his work was published in 1773 [48,49]. Walsh’s experiments probably contributed to Luigi Galvani’s decision to begin his experiments on frog muscle contraction [50]. However, Galvani’s experiments focused on a completely different aspect—the electricity generated by the muscles. Luigi Galvani’s discovery, which took place at the end of the 18th century, is often described as a series of coincidences and experimental observations.
- 1791. Luigi Galvani’s accidental experiment took place in 1791. Galvani was performing anatomical experiments on a frog in his laboratory. At the same time, some of his colleagues were playing with a Leyden jar. While Galvani was working, an electric spark was unexpectedly produced by the Leyden jar. At the exact moment the spark was emitted, Galvani touched the frog’s nerve with a metal knife. The result was a violent contraction of the frog’s muscles, which shocked and fascinated Galvani [34]. The results were published in 1791 [51].
- 1792. George Adams published the paper “An Essay on Electricity: Explaining the Theory and Practice of That Useful Science, and the Mode of Applying it to Medical Purposes” considering the application of electricity in medicine [47].

### 2.3. The 19th Century

In the 19th century, researchers inspired by the previous century continued to investigate electricity, nerve conduction and the study of the strength of the masticatory muscles.

- 1803. Inspired by the work of his uncle Luigi Galvani, Giovanni Aldini was the first to demonstrate the reactions of the muscles of dead men to electrical stimulation from a battery of galvanic cells. His most famous experiment took place at the Royal College of Surgeons in London in 1803, on a hanged man called George Forster [52].
- 1825. Leopoldo Nobili built the astatic galvanometer [53]. An astatic galvanometer is a special type of galvanometer designed to minimize the influence of an external magnetic field on its readings. The main purpose of such a solution is to increase the sensitivity and accuracy of electric current measurements.
- 1838. Inspired by Luigi Galvani, Carlo Matteucci, in an 1838 study, observed the frog’s muscle responses to different types of electrical stimulation, which allowed him to understand the basic mechanisms of bioelectricity in living tissues. He used a complete frog leg, cut off below the knee, with only an isolated nerve above [34]. Carlo Matteucci used the astatic galvanometer invented by Leopoldo Nobili for his research [54]. This discovery was key to understanding the mechanisms by which nerve impulses cause muscle contractions [55].
- 1849. Emil du Bois-Reymond was inspired by the work of Carlo Matteucci. One of du Bois-Reymond’s most important achievements during this period was his demonstration that stimulation of a nerve causes an electrical change, which he called an ‘action potential’. This discovery was fundamental to understanding how nerve impulses transmit information in the body. He described it in 1849 in his work ‘Untersuchungen über thierische Elektricität’ [56]. Du Bois-Reymond also discovered that it was possible to record (using a galvanometer) electrical activity during voluntary muscle contraction [2]. In the same year, Hermann von Helmholtz measured at what speed a nerve signal is transmitted through the nerve fibers of a frog and reported transmission rates ranging from 24.6to 38.4 m per second [57,58].
- 1861. Wilhelm Erb identified specific points on the body that were particularly sensitive to electrical stimulation, known as Erb’s points [59].
- 1862. Guillaume Benjamin Duchenne conducted research into the transcutaneous electrical stimulation of muscles and was able to do so as early as 1833 [34]. However, it was not until 1862 that Duchenne published the work “Mécanisme de la Physionomie Humaine” [60] that he included the results of his research on facial expressions, which he carried out using electrical muscle stimulation, and also included the identification of motor points.
- 1868. Julius Bernstein, in his work from 1868, theorized that a living cell’s interior consists of an electrolytic substance and that the cell is separated from its surrounding environment by a membrane. This membrane is characterized by its limited permeability to ions. Consequently, he suggested that the cell membrane would be the primary resistive component within a cell [61].
- 1870. In the 1870s, Hermann Muller contributed to the classification of the electrical properties of tissues. His work included observing the directionality of the flow of electric charges through biological tissues, such as muscles [47].
- 1890. Étienne-Jules Marey introduced the term “electromyography”. He was the first to record bioelectrical activity [2,62].
- 1895. Greene Vardiman Black conducted a bite force study. He reported that in 1895, he tested the bite force of several thousand people and found that the average was 175 pounds [63]. Black achieved his results by designing a new gnathodynamometer. His device was used to measure intraoral forces caused by vertical jaw movements [64].
- 1898. Napoleon Cybulski proposed the idea that tissue currents could have their own origin. Cybulski concluded that undamaged muscle was the source of the current. Some sources claim that he is the founder of the theory of electric currents in tissues [65].

### 2.4. The 20th Century

The 20th century was critical for the development of surface electromyography. In this period, significant advancements were made in both the theoretical and practical aspects of sEMG. Early in the century, the foundational principles of muscle electricity were established, leading to the development of more sophisticated sEMG equipment. These advancements allowed for the non-invasive recording of muscle electrical activity, providing valuable insights into muscle physiology, movement disorders and rehabilitation. The technological advancements in electronics and computing during the latter half of the century enabled the more precise, real-time analysis of sEMG data, further enhancing its diagnostic and research capabilities. The integration of sEMG with other technologies, like biofeedback and computerized analysis systems, opened new avenues in medical research.

- 1912. Hermann Piper was the first to describe the phenomenon known as the H-wave. The H-wave is the electrical response of a muscle to nerve stimulation. It is a form of reflex that can be recorded by electromyography and its measurement is used to assess peripheral nerve function and neuromuscular integration. Piper analyzed the electrical activity in the muscles in response to electrical stimulation of the ulnar nerve. He published his results in the paper ‘’Elektrophysiologie menschlicher muskeln” [66]. It is worth noting that Hermann Piper is considered to be the first scientist to study the surface electrical signal. This was the first study of surface electromyography. He did this using a string galvanometer [42].

Julius Bernstein published the book “Elektrobiologie”, summarizing his electrophysiological work and expanding his theoretical concepts [67]. This book has made a significant contribution to the field [68].

- 1913–1915. Napoleon Cybulski suggested that the carrier of electricity in skeletal muscle could be ions flowing across the semi-permeable membrane in his work entitled “Prądy czynnościowe nerwów i ich stosunek do temperatur” and in a subsequent work of the same year entitled “Model prądów czynnościowych w mięśniach” [69,70]. In 1915, Napoleon Cybulski recorded the first electrical signal in mammalian (dogs and rabbits) muscle fibers [65,71].
- 1917. Frederick Haven Pratt showed in his work that the amount of energy associated with muscle contraction was due to the recruitment of individual muscle fibers rather than the size of the nerve impulse [72].
- 1919. Gildemeister highlighted the resemblance between the electrical conductivity characteristics of skin and those found in a polarization cell [73].
- 1921. Philippson carried out a significant research project where he measured the electrical impedances of a range of biological substances. This included packed blood cells, muscle and liver tissue from guinea pigs, as well as potato tuber [74].
- 1924. Hans Berger made the first recording of electroencephalography (EEG) in humans and introduced the name that is still used today. He also described the types of waves and rhythms present in a properly functioning brain. This influenced the development of the EMG, sEMG and paved the way for research into human bioelectricity [75].

Hugo Fricke published a technical paper “A Mathematical Treatment of the Electric Conductivity and Capacity of Disperse Systems” that delves into the mathematical analysis of the electrical properties of disperse systems [76]. This work focuses on understanding and calculating the electrical conductivity and capacity of materials composed of dispersed particles or elements. It provides theoretical insights and mathematical models for analyzing these properties, contributing significantly to the field of materials science and electrical engineering.

- 1925. E. K. Liddell and Charles Sherrington made significant contributions to our understanding of motor units in muscle physiology. They conducted experiments on the flexor muscles in the cat’s knee, providing crucial insights into how motor units function. Their work helped to establish the concept of the motor unit, defining it as a single motor neuron and the group of muscle fibers it innervates. This research was fundamental in understanding muscle contraction and the role of the nervous system in controlling muscle movement [77].

In 1925, H. Fricke and S. Morse conducted a study titled “The Electric Resistance and Capacity of Blood for Frequencies between 800 and 4(1/2) Million Cycles”. This research explored the electrical properties of blood, specifically its resistance and capacity over a wide range of frequencies. This study was significant in advancing the understanding of bioelectrical properties of biological materials [78].

- 1929. Edgar Douglas Adrian and Detlev Wulf Bronk developed the concentric needle electrode, which led to the widespread use of electromyography (EMG) [42,79].
- 1937. Joseph Erlanger and Herbert Spencer Gasser published the paper entitled “Electrical Signs of Nervous Activity” [80]. In this work, Gasser and Erlanger described in detail their findings regarding the conduction velocity of nerve impulses and the various properties of nerve fibers. Using a cathode-ray oscilloscope, they were able to accurately record and analyze the electrical signals generated by nerves. Their research contributed to a deeper understanding of the mechanisms of nerve conduction and had a major impact on the development of neurology and electrophysiology.
- 1938. Edmund Jacobson realized that there was a deep connection between muscle tension and emotional state. He conducted numerous experiments using surface electromyography to monitor muscle tension in people in different situations. Through these studies he proved that stress, anxiety and negative emotions lead to an increase in muscle tension. His most important paper was published in 1938—“Progressive Relaxation” [81].

In the same year, Derek Denny-Brown and Pennybacker published the first EMG study in patients with neurological disorders [82].

- 1940. Emergence of surface electromyography [33].
- 1941. Kenneth S. Cole and Robert H. Cole published a research paper “Dispersion and Absorption in Dielectrics I. Alternating Current Characteristics” that delves into the behavior of dielectrics (insulating materials) when subjected to alternating current. It primarily explores how these materials disperse and absorb electrical energy, focusing on their alternating current (AC) characteristics. This study is foundational in understanding the dielectric properties of materials, which has implications in various fields of electrical and materials engineering [83].
- 1943. Graham Weddell published a paper entitled: “Electromyography in Clinical Medicine” [84].
- 1944. V. T. Inman, J B Saunders and L. C. Abbott analyzed the superficial electromyography of shoulder movements [85]. In the same year, Joseph Erlanger and Herbert Spencer Gasser won the Nobel Prize in Physiology or Medicine “for their discoveries relating to the highly differentiated functions of single nerve fibers” [86].
- 1947. Edward Lambert established the first electromyography laboratory in the United States and the first EMG training program. His research began in 1948 and focused on the use of EMG in the diagnosis of myasthenia gravis. Lambert is often referred to as the “father of electromyography” [87]. His early research established EMG as a fundamental neurological tool.

This year also saw the founding of the Dansk Industri Syndikat (DISA), which later developed an analogue electromyograph [88].

- 1948. Price et al. found that back pain affected changes in bioelectrical activity [89].
- 1949. Robert E. Moyers carried out an experiment entitled: “Temporomandibular Muscle Contraction Patterns in Angle Class II, Division 1 Malocclusions; an Electromyographic Analysis” [29]. He was the first to use electromyography to study the masticatory muscles [30].
- 1950. DISA introduces a three-channel analogue EMG recorder (model 13A67). Developed in collaboration with Buchthal at Copenhagen University Hospital [35].
- 1952. Alan Lloyd Hodgkin and Andrew Fielding Huxley published five papers describing a series of experiments and an action potential model [42]. Amongst others, entitled “A Quantitative Description of Membrane Current and its Application to Conduction and Excitation in Nerve”. This work describes in detail how changes in the conductance of sodium and potassium ions across the cell membrane of an axon affect the generation and conduction of an action potentials [90]. Among others, they published another paper entitled: “Currents Carried by Sodium and Potassium Ions through the Membrane of the Giant Axon of Loligo”. This work focused on the study of ionic currents flowing through the membrane of the axon and their role in nerve conduction [91]. Published mainly in the 1950s, it was of great importance to neurophysiology, and its results are still fundamental to understanding the electrical activity of neurons. The Hodgkin–Huxley model is still used today in modern neuroscience research and mathematical models of neuronal activity.

O. C. J. Lippold published a paper entitled: “The relation between integrated action potentials in a human muscle and its isometric tension”. The study explored how the isometric tension produced by a human muscle during voluntary contraction relates to its integrated electromyogram [92].

- 1953. The American Association of Neuromuscular & Electrodiagnostic Medicine was founded [93].
- 1954. Fritz Buchthal published a paper entitled: “Action potential parameters in normal human muscle and their physiological determinants” [94]. This work had a significant impact on the development of electromyography as a diagnostic tool in medicine. Buchthal is therefore considered one of the pioneers of clinical electromyography [71].

Brenda Bigland and O. C. J. Lippold published a paper entitled: “The relation between force, velocity and integrated electrical activity in human muscles” [95]. Among other things, the authors proved that tension, velocity, and electrical activity are closely linked. The integrated electrical record offers a combined measure reflecting both the number of muscle fibers in action and the rate at which they are stimulated [95].

- 1956. Greenfield and Wyke published a paper in which they determine the placement of surface or needle electrodes when studying the masticatory muscles. This is one of the steps in the reproducibility of electromyographic studies [31].

That same year, Joseph R. Jarabak published “An Electromyographic Analysis of Muscular And Temporomandibular Joint Disturbances Due to Imbalances in Occlusion” [96].

- 1959. The American Association of Neuromuscular & Electrodiagnostic Medicine was registered as nonprofit [93].
- 1962. Basmajian, and G. Stecko created a new bipolar electrode for electromyography [97].
- 1963. John Basmajian demonstrated that he could isolate motor units and control their isolated contractions [98].

Peter Vig published the first review paper on electromyography in dental science. In this paper, he described the techniques and their limitations in electromyographic studies of mandibular movements [30].

- 1965. The International Society of Electrophysiological Kinesiology was founded as an international organization. During the International Congress of Anatomy in the Rhein-Maine-Halle, Wiesbaden, Germany in the summer of 1965, a group of anatomists convened to discuss the formation of a small society dedicated to electrophysiological kinesiology. Among those present were notable individuals like J.V. Basmajian, S. Carlsøø, B. Jonsson, M.A. MacConaill, J. Pauly and L. Scheving [99].

In the same year, Elwood Henneman, George Somjen and David O. Carpenter published a paper entitled: “Functional Significance Of Cell Size In Spinal Motoneurons” [100]. They published a further four studies describing in detail the activation properties of motor units or motoneurons. Their findings can be summarized in a single thesis, which they called the “size principle”. This thesis states that the smaller motoneurons in the anterior horn of the spinal cord are likely to correspond to the smallest motor units [42,100].

- 1966. Moller published a paper entitled: “The chewing apparatus: An electromyographic study of the action of the muscles of mastication and its correlation to facial morphology”. It was one of the first papers of its kind on the action of the masticatory muscles and its correlation to facial morphology [101].
- 1968. Herman P. Schwan published a paper entitled: ‘’Electrode Polarization Impedance And Measurements In Biological Materials” [102]. It appeared in the Annals of the New York Academy of Sciences. This study is significant in the field of bioimpedance, particularly focusing on the challenges and methodologies related to measuring the electrical properties of biological materials.
- 1969. R. Yemm conducted some of the first more sophisticated research on the bioelectrical activity of the human masseter muscle associated with emotional stress [103]. In the following years, Yemm continued his research on the above topics.

J. M. Bernstein, N. D. Mohl and H. Spiller published papers on temporomandibular joint dysfunction and its symptoms manifested as diseases of the ear, nose and throat [104].

In the same year, E Moller published a paper entitled “Clinical electromyography in dentistry” [105]. The paper presents electromyography as a diagnostic method in dentistry.

- 1971. C. J. Griffin and R. R. Munro published papers on the direct effect of TMDs on the electrical activity of the masticatory muscles [106].
- 1975. DISA produced the first complete digital EMG system and all modules are equipped with digital components (Model 1500) [35].

In the same year, H. S. Milner-Brown and R. B. Stein published a paper entitled: “The relation between the surface electromyogram and muscular force” [107]. This work focuses on the study of motor units in the first dorsal interosseous muscle of the healthy subject. It explore how the wave form contributed by each motor unit to the sEMG is determined and relates to muscle force. This includes an analysis of the amplitude and duration of the wave forms and their relationship to muscle recruitment and force levels. The study discussed the distribution of motor units in the muscle and the relation of sEMG to muscle force production [107].

- 1978. G. J. Pruim, J. J. Ten Bosch and H. J. de Jongh published a paper entitled: ‘’Jaw muscle EMG-activity and static loading of the mandible” [108]. The study outlines a method for correlating EMG activity in jaw muscles with static biting forces. It supports the linear relationship between integrated EMG activity and force produced by muscles in isometric conditions. It also notes distinct functional behaviors in the anterior and posterior parts of the temporal muscle. The study emphasizes the significant role of opening muscles as antagonists, suggesting their importance should not be overlooked in muscle force analysis [108].
- 1979. Bernard Jankelson, with an international group of clinicians and dental educators, founded the International College of Craniomandibular Orthopedics (ICCMO). Bernard Jankelson’s motto is “If you can measure it is a fact. If you cannot measure it is an opinion” [109].
- 1980. M. Bakke and E. Møller conducted a study entitled ‘’Distortion of maximal elevator activity by unilateral premature tooth contact” [110]. On the basis of this, they note that a one-sided premature contact resulted in a notable imbalance of action across all the muscles being examined, showing increased activity on the side of the contact. As the thickness of the overlay was enhanced, there was a corresponding decrease in the mean voltage on both sides. This asymmetry is thought to be due to heightened spindle afferent activity on the side of the contact compared to the opposite side. Furthermore, the overall decrease in muscle activity is attributed to a progressive reduction in activity stemming from the periodontal pressure receptors [110].
- 1981. M. Yardin studied the effect of head displacement on the change in electrical activity of the masticatory muscles [111].
- 1982. InterSoft introduced the first fully computerized EMG system, the package included motor unit analysis and interference pattern analysis [35].

In the same year, Erik Stålberg and Lars Antoni developed a mini-computerized EMG system. The software allowed, among other things, analysis of motor units based on template matching and scanning EMG [112].

In the same year, C. Riise and A. Sheikholeslam proved that the anterior temporal muscles and sometimes the masseter muscles exhibit postural activity. Additionally, it appears that occlusal interferences, similar to those commonly created in routine dental treatments like fillings, crowns, and bridges, can influence the neuromuscular pattern of the mandibular elevators when they are at rest. This impact is observed in the context of occlusal rehabilitation practices [113].

**Figure 3:**
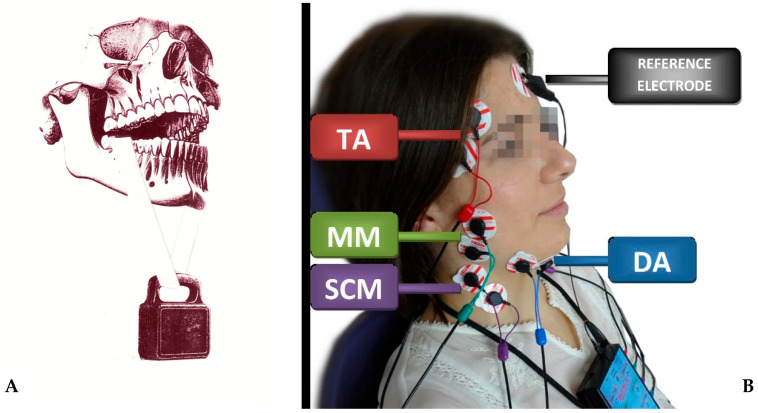
The difference of 340 years of research on masticatory muscles between the discoveries of Giovanni Alfonso Borelli and modern sEMG studies (8-channel BioEMG III electromyography, BioResearch Associates, Inc., Milwaukee, WI, USA). (**A**) shows the assumptions of the 1681 Giovanni Alfonso Borelli study and (**B**) the 2021 study. TA—the temporalis anterior muscle; MM—the superficial part of the masseter muscle; DA—the anterior belly of the digastric muscle; SCM—the middle part of the sternocleidomastoid muscle. The elements for graphic A were generated on 2.01.2024 using the DALL-E 2 program (OpenAI, Inc., San Francisco, CA, USA), and merged by the author G.Z.

- 1983. S. E. Widmalm and S. G. Ericsson observed changes in the bioelectrical activity of the masticatory muscles in response to a visual stimulus [114].
- 1984. Robert Jankelson and Mary Lynn Pulley published the textbook ‘’Electromyography in Clinical Dentistry’’ [115].
- 1985. BIOPAC (BioResearch Associates, Inc., Milwaukee, WI, USA) was founded, a company that manufactures equipment for electromyographic testing, including sEMG of the masticatory muscles [116] (Figure 3).
- 1986. A. Sheikholeslam, K. Holmgren, and C. Riise suggested that using an occlusal splint can alleviate or reduce the signs and symptoms of functional disorders. It can also normalize and lessen the postural activity in the temporal and masseter muscles. This improvement may aid in procedures like functional analysis and occlusal adjustment by establishing more balanced muscle activity [117].
- 1989. M. Naeije, R. S. Mccarroll, W. A. Weijs created activity and asymmetry patterns for the masseter and the anterior temporal muscles [118].
- The 1980s/1990s. Noraxon, a company developing software for biomechanical testing, including electromyography, is founded. It is currently the most advanced equipment for sEMG testing [119].
- 1990. Jeffrey R. Cram published the book “Clinical EMG for Surface Recordings”, which focuses on the clinical use of surface electromyography [120].
- 1991. The group of V.F. Ferrario, C. Sforza, A. D’addona, A. Miani Jr., using the BIO-PAK system (Bio-Research Associates Inc., Milwaukee, WI, USA), stated that the electromyographic system and protocol used allowed good reproducibility of measurements in chewing muscle testing. The authors stated that electromyographic testing has potential applications in clinical and research settings [121].
- 1996. Under the leadership of Dick Stegeman, the development of the HD-sEMG technique (High-Density Surface Electromyography) took place. This is a more sophisticated version of classical surface electromyography (sEMG), with more electrodes and higher spatial resolution [42].
- 1998. Jeffrey R. Cram produced another textbook on electromyography entitled “Introduction To Surface Electromyography” [122].

### 2.5. The Beginning of the 21st Century 

The 21st century has brought significant advancements in sEMG. One of the key developments has been the development of advanced signal processing algorithms that enable more accurate analysis of muscle activity. This has made it possible to accurately distinguish the activity patterns of individual muscle fibers, which is important for diagnosis and rehabilitation. Another important step has been the miniaturization and improved portability of sEMG devices, allowing them to be used in natural environments, such as during daily activities or sports. In addition, developments in wireless technology and telemetry have enabled remote monitoring and real-time data analysis.

**Figure 4:**
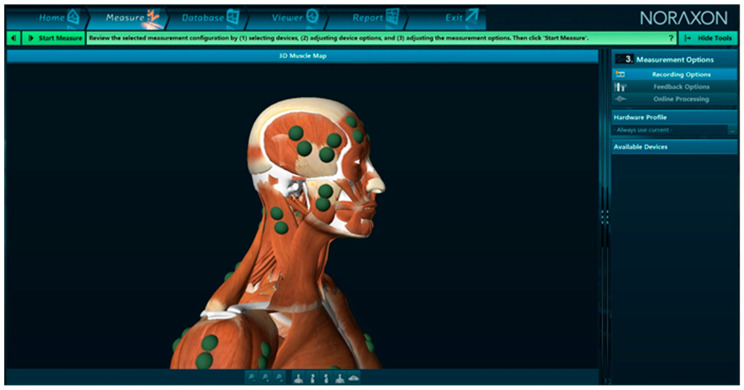
Graphic showing the position of the temporalis and masseter muscle electrodes according to the SENIAM program. Screen from Noraxon MR3 3.18.08 software (Noraxon USA, Inc., Scottsdale, AZ, USA).

- 2000. H. J. Hermens, B. Freriks, C. Disselhorst-Klug and G. Rau published a SENIAM program. The SENIAM project was organized as a European joint action, funded in the context of the European Community’s Biomed 2 program (1996–1999) [32] (Figure 4).

“The objectives of the project were to integrate basic and applied research on surface EMG (sEMG) at a European level to establish European co-operation and to solve key items that presently prevent a useful exchange of data and clinical experience. The key topics of this joint effort were (1) sEMG sensors and sensor placement procedures, (2) sEMG signal processing and (3) sEMG modeling” [123].

In the same year, the group of V. F. Ferrario, C. Sforza, A. Colombo, and V. Ciusa highlighted the role of electromyographic signal standardization in dentistry [124].

- 2005. Peter Konrad created a textbook for sEMG studies entitled: “The ABC of EMG” [1].
- 2007. Gea Drost created the textbook “High-density surface EMG—pathophysiological insights and clinical applications” [42].

The history of sEMG in dentistry relates to the origins of anatomy, the search for what moves tissue, the discovery of electricity, detection and analysis of tissue bioimpedance and the history of dental research. The basis was the understanding of the “forces” that move muscles, the discovery of electricity. Furthermore, the 20th century proved to be crucial for the field of SEMG in dentistry. For example, sEMG for the analysis of the masticatory muscles was used in 1949 by Robert E. Moyers [29]. He was the first to use electromyography to study the masticatory muscles [30]. In 1956, Greenfield and Wyke published a paper in which they determined the placement of surface or needle electrodes when studying the masticatory muscles. This is one of the steps in the reproducibility of electromyographic studies [31]. In 1963, Peter Vig published the first review paper on electromyography in dental science. In this paper, he described the techniques and their limitations in electromyographic studies of mandibular movements [30]. The beginning of the 21st century was associated with the standardization of the sEMG examination (e.g., the SENIAM protocol [32] and the activities of the Ferrario et al. group [124]). History shows that there is a systematic drive to create ever smaller, more accurate and more mobile sEMG instruments.

## 3. Surface Electromyography in Dentistry—Present

**Figure 5:**
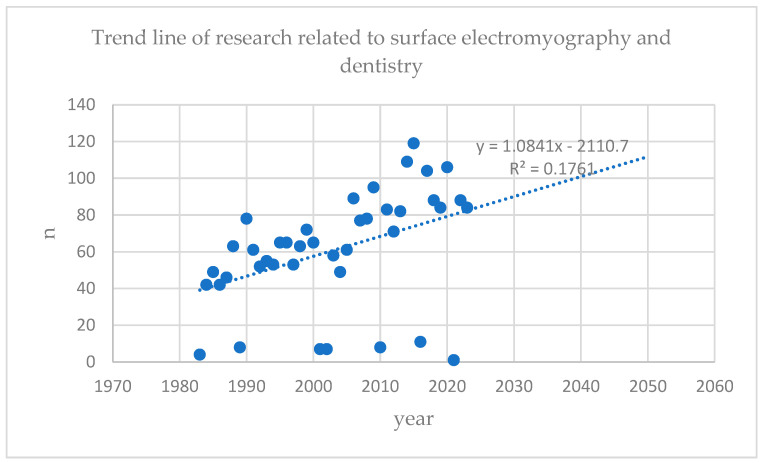
Publication trend line of studies using surface electromyography in dentistry. We would like to point out that the above data are illustrative, showing some of the familiarity of sEMG research in dentistry. The authors do not always use these specific keywords (“surface electromyography” AND “dentistry”).

It has been 60 years since the review by P. Vig (“Electromyography In Dental Science: A Review”) [30] and 74 years since the first use of sEMG in dentistry (until 2023) [29]. Actually (as of 5 January 2024), there are 3136 papers in the PubMed database related to the keywords “surface electromyography” AND “dentistry”. These 3136 papers include (according to the PubMed bay filters) 211 clinical trials, 7 meta-analyses, 131 randomized controlled trials, 133 reviews, 32 systematic reviews. Based on the data obtained from (one of the more recognized databases) the PubMed database, trend lines were drawn suggesting a systematic increase in electromyographic studies in dentistry (Figure 5).

Modern sEMG studies in dentistry represent a significant step forward in the diagnosis and treatment of conditions related to the stomatognathic system. In addition to what has already been described in the introduction, i.e., the use of sEMG for assessing muscle activity during physiological and parafunctional activities [6], it is also used in the diagnosis of temporomandibular joint and masticatory muscle function and treatment of the TMDs [7,8,9,10]. Studies on the influence of psychological [11,12,13,14] and physical state on changes in the bioelectrical activity of the masticatory muscles appear on the sEMG [15,16,17,18]. 

At the beginning of the second decade of the 21st century, sEMG has been used, among other things, to assess the effect of wearing a mask on the masticatory muscles [125,126], the effects of physical activity on the masticatory muscles [127,128,129,130], and the effects of botulinum toxin on the masticatory muscles [131].

In addition, new Functional Indices of Masticatory Muscle Activity were developed by Ginszt and Zieliński [132], which can be used in addition to Asymmetry and Activity Indices [118], the Percentage Overlapping Coefficient (POC), the torque coefficient (TC), and Maximum Voluntary Contraction (MVC) [124] used in electromyographic tests in dentistry.

### 3.1. Publication Trends in the PubMed Database in 2023 under the Keywords “Surface Electromyography” and “Dentistry”

Acute trends in surface electromyography research in dentistry in 2023 focus on

- Studies in patients with TMDs [7,133,134];
- Studies in patients with bruxism [135,136,137,138,139,140];
- Investigations in patients with other conditions (e.g., age-related, myofunctional disorders, cleft lip/palate, and oral parafunction) [141,142,143,144,145];
- Treatment and dental rehabilitation [146,147,148,149,150,151,152,153,154];
- Examination of occlusal conditions [155,156,157,158,159,160,161,162,163];
- Verification of new electromyographs [164,165].

### 3.2. Apparatus for Surface Electromyography in Dental Research in the 21st Century

**Figure 6:**
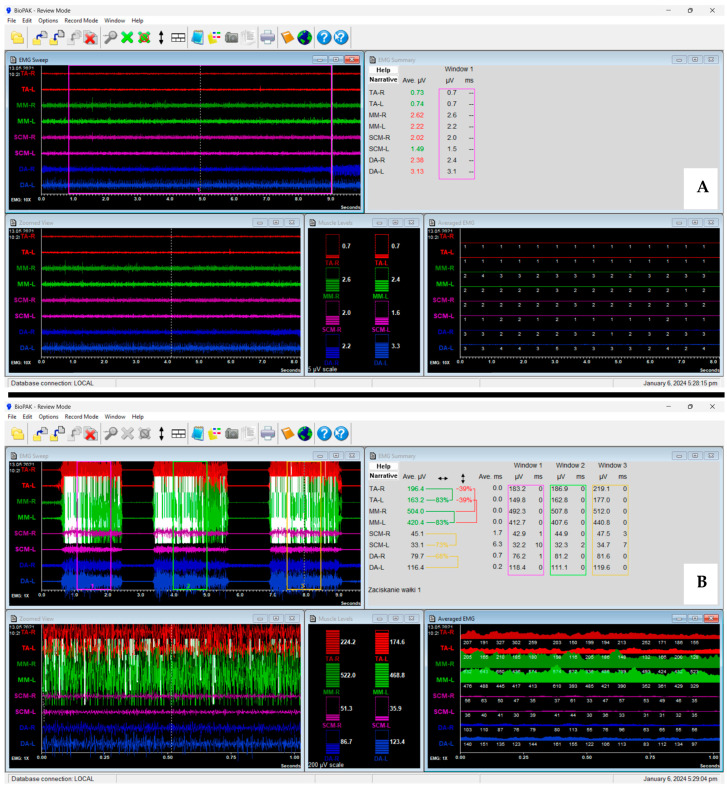
Screen from BioPAK software (version 8.8). (**A**)—during resting mandibular position; (**B**)—during the maximum voluntary clenching on dental cotton rollers in the intercuspal position. TA—the temporalis anterior muscle; MM—the superficial part of the masseter muscle; DA—the anterior belly of the digastric muscle; SCM—the middle part of the sternocleidomastoid muscle; R—right side; L—left side.

The most commonly used apparatus for surface electromyography is the eight-channel electromyograph BioEMG III™ (Figure 3B) compatible with the BioPAK measurement system (Figure 6) (BioResearch Associates, Inc. Associates, Inc., Milwaukee, WI, USA) [17,145,154,155,159,160].

The BioEMG III™ electromyograph records electrical (bio-potential) activity from eight muscles simultaneously (right and left side of the temporalis anterior muscle, the superficial part of the masseter muscle, the anterior belly of the digastric muscle, and the middle part of the sternocleidomastoid muscle). Microvolt signals are amplified, virtually without noise, to 5000 times their original levels [166]. 

The amplifier has an impedance greater than 100 MegOhms, precisely 108 MegOhms. It can handle input signals in the range of 0 to 2000 microvolts (peak to peak). The amplifier has low noise, producing less than 0.3 microvolts on average. The DC offset range of the amplifier is from −0.7 volt to +0.7 volt. It exhibits excellent common mode rejection, with a ratio of greater than 130 dB at 60 Hertz and greater than 120 dB from 100 to 600 Hertz. The maximum signal to noise ratio is an 1,000,000 to 1. It can handle common mode voltages ranging from −6.5 to +6.5 volts DC. The amplifier has a bandwidth of 30 to 1000 Hertz when operating at a 2000 Hertz sample rate. Sensitivity is less than 0.3 microvolts (peak to peak). The resolution of the A/D converter is 0.5 microvolts. It provides silent period measurements to the nearest millisecond. It can withstand a maximum peak voltage of 3500 volts when applied. The amplifier has low leakage current, measuring less than 1.0 microampere. The capacitance to the AC power line is minimal, less than 2 picofarads. Software Noise Reduction is an essential feature in various electronic devices and equipment, including electromyographs like the BioEMG III. In this context, the BioEMG III electromyograph incorporates Software Noise Reduction, with a nominal noise reduction of 40 dB. To put this in perspective, a reduction of 40 dB on a logarithmic scale translates to an impressive 99% reduction in noise amplitude when viewed on a linear scale. This means that the device effectively minimizes unwanted noise, ensuring that the recorded signals are exceptionally clean and accurate for precise analysis and diagnosis [166,167].

The amplifier incorporates Underwriters Laboratories recognized opto-couplers for electrical isolation, ensuring the patient is separated from the computer’s AC power supply. These opto-couplers have Underwriters Laboratories file numbers E58730, E54915, or equivalent. Signals are displayed on a computer as original time domain wave forms and averaged levels, revealing contraction patterns and relative intensities [166,167]. BioEMG III™ has the disadvantage that it must be connected to the laptop at all times during signal recording.

**Figure 7:**
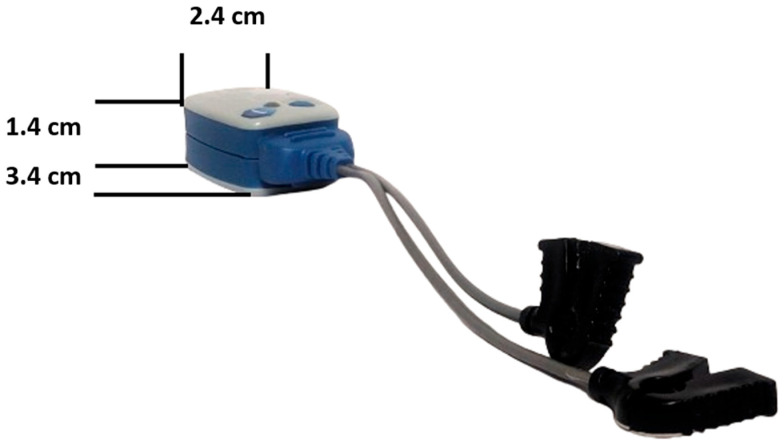
Modern sEMG sensor along with its dimensions (Ultium EMG, Noraxon USA, Inc., Scottsdale, AZ, USA). The first electromyographic machines were stationary and larger than 1 m in size [34,35]. Today’s SEMG machines are fully mobile and small, with modules that allow wireless examination.

One form of wireless sensor is the Ultium EMG, which is compatible with myoMOTION™ (MR 3.20.62 software, Noraxon USA, Inc., Scottsdale, Arizona, USA) [168,169] (Figure 7). This wireless transmission idea significantly streamlines the process of gathering sEMG measurements by eliminating the need for cable connections between the sEMG electrodes and sEMG amplifier. The Ultium System is engineered to function with any setup ranging from 1 to 16 sensors (with the option to use up to 32 sensors simultaneously). Additionally, the sEMG sensors can be activated to record inertial measurement unit data, or a combination of sEMG and acceleration data can be collected. The sEMG sensor’s transmission range typically extends up to 40 m [170].

The system features a high-resolution data acquisition system with 16-bit precision. Users can select between two sample rates, 2000 Hz or 4000 Hz, for data acquisition. The device does not employ notch (50/60 Hz) filters. It offers selectable high-pass filter cutoff options of 5 Hz, 10 Hz, or 20 Hz. Users can also choose between low-pass filter cutoff frequencies of 500 Hz, 1000 Hz, or 1500 Hz. The internal sampling resolution is 24 bits. The baseline noise level is less than 1 microvolt root mean square (RMS). The input impedance is greater than 100 MegOhms. It boasts a common mode rejection ratio exceeding 100 dB. The input range spans from ±24,000 uV. Adaptive resolution is applied: below ±5000 microvolts, it achieves 0.3 microvolts, while above ±5000 microvolts, it reaches 1.16 microvolts. The device offers a measurement functional accuracy of ±1% of the full scale. Analog output is scaled either as 24,000 microvolts/5 V or 4800 uV/1 V. A fully charged battery allows the sensor to operate for up to 8 h, with a recharge time of 3 h [169,170].

The model 810 EMG Sensor has obtained clearance for market distribution within the European Community through certification from Notified Body #2797, which is the British Standards Institution. Furthermore, this device conforms to the international diagnostic EMG standard (IEC 60601-2-40) [170]. The Ultium EMG (Noraxon USA, Inc., Scottsdale, AZ, USA) is also used in the studies of masticatory muscles [128,171,172,173]. It is worth noting that this sEMG can examine different muscles of the body and is not dedicated to a specific group [170]. 

### 3.3. The Future of Surface Electromyography in Dental Research

The history described in this paper shows the continuous improvement in surface electromyographs. As improvements have been made and their size has become smaller, reliability and accuracy have increased. It is likely that future electromyographs will use smaller, more discreet sensors to record muscle activity. These devices will incorporate wireless technology, allowing real-time data transmission to a nearby monitoring system or mobile app for instant analysis and feedback. For example, in the context of research into bruxism, this will be crucial for patient treatment. Artificial intelligence is likely to be used to analyze the data.

The reality of the above description may be due to the fact that the technology has steadily become more sophisticated over the years [174,175]: the original sEMG were not mobile, whereas the current ones are, and modern sensors are not only mobile, but the transmission range of the sEMG sensor is up to 40 m [35,170]. Research has already been published demonstrating the feasibility of analyzing masticatory muscle activity using smartphones [163,176].

When it comes to artificial intelligence, it has already been pointed out that it will revolutionize healthcare [177,178]. Moawad et al. note that the deep learning component of artificial intelligence can be used for quality control, workflow and reporting [178]. This is a normal trend of medical informatics which involves the adaptation of new technologies in a medical context [179].

## 4. Surface Electromyography in Dentistry—Recommendations for the Methodology

Reproducibility in medical research refers to the consistency and reliability of study outcomes when the same experiments or clinical investigations are repeated multiple times [180]. High reproducibility is essential to validate findings, ensure the accuracy of diagnostic tests, and establish the effectiveness of medical treatments. Factors influencing reproducibility include standardized methodologies, consistent data collection, sample size, and minimizing potential biases [181,182]. Ensuring reproducibility in medical research helps enhance patient care, clinical guidelines, and the overall reliability of healthcare practices [182].

To ensure high reproducibility of sEMG examinations, it is important to describe in detail the preparation of the skin for the test, the location of the electrodes, the position of the subject’s body, the test procedures, sEMG equipment specifications and signal processing information.

Skin preparation is described in Konrad’s paper [1]; electrode placement should be according to the SENIAM program [32], and the subject’s body position is usually described in studies. In most dental studies, this is the sitting position [17,153,158,160]. Examination procedures vary according to the purpose of the study. However, there should undoubtedly be a standardization of procedures when examining the masticatory muscles. Typically, the sEMG study of the resting mandibular position includes 10 s (s) of rest [17,145,153,172]. However, the maximum voluntary clenching in the intercuspal position is reported differently:

- Two clenches of 3 s each with a 3 s break [124,146],
- Two clenches of 3 s each with a 30 s break [137],
- Two clenches of 3 s each with a 180 s break [158],
- Three clenches of 3 s each with a 2 s break [145,153,159],
- Three clenches of 5 s each with a 60 s break [147].

It is worth noting that studies do not always include information on equipment specifications such as sample rates, bandwidth, input impedance of the sEMG used, common mode rejection ratio, input range or baseline noise [135,136,140,157,158,159,165,172]. 

Reproducibility of sEMG studies must be based on precise specifications, especially as modern electromyograms have the ability to adjust the above parameters according to the examiner’s settings. Without this information, there can be no talk of reproducibility.

### 4.1. Sample Rates 

Compliance with the Nyquist–Shannon theorem: In order to reproduce a signal correctly, the sampling frequency must be at least twice the highest frequency of the signal [183,184]. For sEMG, which records electrical signals generated by muscles, this is particularly important, as muscles can generate signals at different frequencies [185]. A higher sampling rate allows more accurate recording of changes in the signal, which is essential for accurate analysis of muscle function. Based on current sEMGs, the appropriate choice of sampling rate for muscle may be 2000 Hz or higher to ensure sufficient accuracy [166,167,170].

### 4.2. Bandwidth

Bandwidth is one of the key parameters in sEMG research. Bandwidth determines the range of frequencies effectively recorded by the sEMG system [186]. Muscles generate electrical signals at different frequencies depending on the type of muscle fiber, the type of muscle activity and other factors. To obtain accurate and representative data, the sEMG system must be able to capture the full range of relevant frequencies.

The sEMG signal contains information about muscle activity in different frequency ranges. Low frequencies (below 20 Hz) can reflect slower, more tonic muscle activity, while higher frequencies (500 Hz and above) represent faster, more phasic muscle activity [187,188,189]. An appropriate bandwidth allows these different aspects of muscle activity to be captured effectively. By setting the appropriate bandwidth, external and internal noise unrelated to muscle activity can be eliminated. For example, a low-pass filter removes high-frequency interference such as electrical noise, and a high-pass filter reduces slow signal fluctuations that can be caused by body movement or changes in electrode position [190].

The study by Karacan et al. 2023 highlighted the importance of using filters in sEMG studies [191]. For the masticatory muscles in sEMG studies, the recommended frequency limits are usually between 20 Hz and 500 Hz [10,192,193].

### 4.3. The Impedance of the Electrodes and the Input Impedance of the sEMG Used

Input impedance refers to the electrical resistance that the sEMG device places on the flow of current from the electrodes [194]. A high input impedance is critical as it minimizes signal distortion and allows for more accurate readings. A low input impedance can cause signal distortion and affect the accuracy of measurements [195]. For sEMG applications for masticatory muscles, a high input impedance is recommended, often exceeding 100 MegOhms [17,116,169,170,196]. Konrad describes that the input impedance of the amplifier should be at least 10 times the impedance of the electrode [1].

### 4.4. Common Mode Rejection Ratio 

Common mode rejection ratio is a measure of an amplifier’s ability to reject signals that are common to both inputs [1,197]. In practice, this means that the amplifier is better at separating the desired signal from interference, such as electrical noise or other common environmental noise [195]. A common mode rejection ratio greater than 100 dB appears to be most appropriate for musculoskeletal studies [198,199,200]. Such a value indicates a good ability of the device to reject common mode interference for both electrodes, which is critical in environments with a lot of potential electrical interference.

### 4.5. Input Range

The input range defines the maximum signal amplitude that the device can effectively process without distortion. The input range should be wide enough to accommodate the expected amplitudes of the sEMG signals, which may vary depending on the muscles being tested [196,201]. 

### 4.6. Baseline Noise

Baseline noise refers to the electrical background noise of the device when it is not recording muscle activity [202]. Konrad defines the ideal baseline noise as 1–2 millivolts [1].

### 4.7. Signal Processing

For example, the BioPAK measurement system has automatic signal processing, while the system with myoMOTION™ software will require the selection of appropriate filters [116,168,170]. It is important to state directly in the paper how the signal was processed, whether automatically or by the researcher selecting the exact filters. If filters were selected, it should be stated which ones and in what order.

In the sEMG of the masticatory muscles, standard signal processing often includes rectifying the sEMG signal and calculating the smoothed RMS value [1,203].

### 4.8. Main Recommendation for sEMG

Our main recommendation is to describe in detail all the parameters of the sEMG process (especially the parameters of the equipment used and how the signal is processed). To ensure that researchers do not overlook any important element, a checklist has been provided in (Supplementary Materials Table S1).

### 4.9. Implications for Practice and Research—Recommendation for sEMG

Accurately describing study procedures, including electrode placement, measurement protocols and analysis methods, helps ensure the reliability of the results obtained. This is crucial in research as it allows other researchers to reproduce studies and verify results. Accurately describing test procedures promotes the standardization of measurement methods in the field of electromyography. This makes it possible to compare results between different international studies and facilitates progress in the field. In the historical section, we have shown that this is what researchers have been striving for in terms of reproducibility of results [1,121,123,124].

In the context of clinical applications, the accurate description of sEMG studies can be important for diagnosis and treatment planning. With precise examination protocols, EMG data can be used more effectively in clinical practice. This leads to improved standards of diagnosis and treatment in dental examinations. However, in order to have a significant clinical impact on the therapeutic process, it is essential that examinations are performed correctly (correct setting of electromyographic parameters). Therefore, it is important to standardize electromyographic parameters [7,19].

## 5. Conclusions

- sEMG is a diagnostic technique that has found significant application in dentistry, particularly in the study of physiological function and masticatory muscle function.
- sEMG equipment continues to evolve into smaller and more precise systems.
- The increased technical capabilities of sEMG equipment and the ability to specify parameters (e.g., sampling rates, bandwidth) demonstrate the need for a detailed description of selected parameters in the methodological section. This is necessary to maintain the reproducibility of sEMG testing.
- More high-quality clinical trials are needed in the future.

## Supplementary Materials

The following supporting information can be downloaded at: https://www.mdpi.com/article/10.3390/jcm13051328/s1, Table S1. Recommendations for a list of elements that should be included in the description of the sEMG study.
