# Supplementary material for: Surface Electromyography in Dentistry—Past, Present and Future
Source: J Clin Med. 2024 Feb 26;13(5):1328. doi: 10.3390/jcm13051328 (PMC10931581; doi:10.3390/jcm13051328)
Supplement: Supplementary file 1 [file jcm-13-01328-s001.zip › jcm-2869449-supplementary.pdf]

Table S1. Recommendations for a list of elements that should be included in the description of the sEMG study.

No.		Has been described?
1.	How was the skin prepared for the study?	☐
1.1.	What was used to cleanse the skin and how?	☐
1.2.	During what hours was the sEMG study conducted?	☐
2.	How were the electrodes positioned?	☐
2.1.	Was the placement in accordance with the SENIAM program guidelines?	☐
2.2.	What kind of electrodes were used (what was the conductive surface area, and the specific manufacturer)?	☐
2.3.	What was the maximum acceptable electrode impedance in the study?	☐
3.	What was the subject's position during the sEMG study?	☐
4.	What was the research procedure like?	☐
4.1.	What activities were performed by the subject during the study?	☐
4.2.	How many repetitions were performed (what were the intervals between repetitions)?	☐
5.	What sEMG equipment was used, and the specific manufacturer?	☐
5.1.	What was the sample rate?	☐
5.2.	What was the bandwidth (high-pass filter and low-pass filter cutoff frequencies)?	☐
5.3.	What is the input impedance of the used sEMG?	☐
5.4.	What was the common mode rejection ratio?	☐
5.5.	What was the input range?	☐
5.6.	What was the baseline noise?	☐
5.7.	What were the other important details about the device?	☐
6.	How was the signal processed?	☐
6.1.	Was there automatic processing?	☐
6.1.1.	If yes, was the exact name of the program and distributor given?	☐
6.1.2.	If no:	
6.1.2.1.	what filters were used?	☐
6.1.2.2.	in what order were the filters used?	☐
6.1.2.3.	what software was used for this purpose (name of the software and the specific manufacturer)?	☐
